# Biomonitoring of manganese metal pollution in water and its impacts on biological activities of *Biomphalaria alexandrina* snail and larvicidal potencies

**DOI:** 10.1007/s11356-023-29786-x

**Published:** 2023-09-18

**Authors:** Amina Mohamed Ibrahim, Ahmed Abdel-Salam Abdel-Haleem, Rania Gamal Taha

**Affiliations:** 1 Medical Malacology Department, Theodor Bilharz Research Institute, Imbaba, Giza Egypt; 2 Biological and Geological Sciences Department, Faculty of Education, Ain Shams University, Roxy, Heliopolis, Cairo, P.C.11757 Egypt

**Keywords:** Manganese metal, *B. alexandrina*, Bioaccumulation factor, Larvicidal, Hormonal alterations, Histopathology

## Abstract

Metal pollution has many dangerous environmental and human health consequences due to the bioaccumulation in the tissues. The present study aims to measure the bioaccumulation factor of the manganese (Mn) heavy metal in *Biomphalaria alexandrina* snails’ tissues and water samples. The current results showed the concentration of Mn heavy metal in water (87.5 mg/l) and its bioaccumulation factor in *Helisoma duryi* tissue was higher than that in tissues of *Physa acuta* and *B. alexandrina* snails. Results showed that 87.5 mg/l Mn concentration had miracidicidal and cercaricidal activities. Also, this concentration decreased the mean total number of the hemocytes after exposure for 24 h or 48 h, while increasing both the mean mortality and phagocytic indices of the hemocytes of exposed snails. It caused alterations in the cytomorphology of the hemocytes of exposed snails after 24 or 48 h, where the granulocytes had irregular cell membranes and formed pseudopodia. Besides, levels of testosterone (T) and estradiol (E) were increased after exposure to 87.5 mg/l Mn metal compared to the control group. Also, it increased MDA (malonaldehyde) and TAC (total antioxidant capacity) contents, while decreasing SOD (superoxide dismutase). Besides, it caused significant histopathological damages in both hermaphrodite and digestive glands, represented in the degeneration of the gonadal, digestive, secretory cells, and the connective tissues. Therefore, *B. alexandrina* might be used as a sensitive bioindicator of pollution with Mn heavy metal to avoid ethics rules; besides, they are readily available and large in number.

## Introduction

Environmental metal pollution represents a dramatic problem in the world (Salih et al. 2021). The heavy metals are natural parts of the earth’s crust, but higher levels of these metals could threaten the environment and the biological system of human (Ugbaja et al. 2020; Briffa et al. 2020). Since the 1940s, the rate of these heavy metal was increased worldwide due to industrialization and urbanization (Ali et al. 2019).

Heavy metals were classified as dangerous pollutants as they bioaccumulated in the organisms’ bodies after swallowed or inhaled causing biological and physiological complications (Briffa et al. 2020). Some heavy metals are essential elements for life with small traces (Ni, Cu, Zn, and Cr) (Dhiman and Pant 2021). Heavy metals, such as manganese (Mn), zinc (Zn), nickel (Ni), copper (Cu), cadmium (Cd), chromium (Cr), lead (Pb), and iron (Fe), have high levels of toxicity and ability for bioaccumulation and combination into the aquatic food chain subsequently reaching in the aquatic ecosystems (Kibria et al. 2016). Heavy metal pollution of the aquatic environment caused severe damage to fish tissues and hence can affect human health (Ali et al. 2019). Manganese (Mn) is an essential metal required for enzymes and cofactors, but its higher concentration could cause several neurological disorders (Schmidt and Husted 2019).

Environmental biomonitoring could help to elucidate the harmful effects of the heavy metal on the biological systems of the surrounding organisms (Morais et al. 2022). Bioindicators are a group of species that reflected the natural state of the environment including biotic or abiotic changes in the habitat (McGeogh 1998). Several aquatic species have been used as bioindicators for chemical pollution (Ibrahim and Sayed 2019; Hamdi et al. 2021). Mollusks are widely used as bioindicators and biosensors of heavy metal poisoning due to their rapid accumulation of these toxic pollutants, widespread and have a moderate life span (Abd-Allah et al. 1997; Ugbaja et al. 2020). These aquatic invertebrates could replace the higher vertebrate in ecological studies because of the ethical rules and the low cost (Ibrahim et al. 2018; Dhiman and Pant 2021).

*Biomphalaria alexandrina* snails the intermediate host for *Schistosoma mansoni* in Egypt (Abdel-Ghaffar et al. 2016; Ibrahim and Abdalla 2017) are widely distributed throughout the Nile River (Ibrahim and Sayed 2019; Morad et al. 2022), where schistosomiasis can affect at least 236.6 million people in 2019, as it causes neurotoxicity, nephrotoxicity, and hepatotoxicity (Dkhil et al. 2016; WHO 2022). *Biomphalaria alexandrina* can serve as a good and sensitive bioindicator of heavy metal pollution (Habib et al. 2016). These snails proved to be a good bioindicator because of their adult and embryonic phases could reflect the pollution by both physical and chemical agents (Morais et al. 2022).

Therefore, the objective of the present study is to measure the concentration of the manganese heavy metal in water samples and *B. alexandrina* tissues and calculation of the bioaccumulation factor and then study its concentration in water on survival and infection rates of *B. alexandrina* with *Schistosoma mansoni* miracidia and its cercaricidal activities and also to elucidate its immunotoxicity, endocrine disruption, oxidative stress biomarkers, and histopathological effects on tissues of these snails.

## Materials and methods

### Field study

#### Snail samples and water collection

Snails were collected from different irrigation canals in Abu Rawash, Giza Governorate. It was placed in numbered plastic aquaria with water from these canals. Also, water samples were collected in sterilized 1-liter polyethylene bottles below the water’s surface of about 30 cm (Kaufmann et al. 1988). Both samples were transported in an ice box to the laboratory for analysis within 12 h.

#### Estimation of heavy metals in *B. alexandrina* snails’ soft tissue and water samples

Determination of heavy metals in water samples and snail’s tissues was performed by using atomic absorption spectrophotometry (AAS) in Environmental Research Laboratory, Theodor Bilharz Research Institute (TBRI), according to the method of Abdel Kader et al. (2016).

1. Analysis of heavy metals in water: For the analysis of total heavy metals, water samples (200 ml) were digested with 5 ml of acidified concentrated nitric acid (HNO3) on a hot plate and filtered by Whatman No. 42 filter paper and made up the volume to 50 ml by double distilled (ddH_2_O) (Shaaban et al. 2017).
2. Analysis of heavy metals in *B. alexandrina* snails’ tissues: About 0.01 g of the snail’s soft tissues were separated from their shells, oven-dried at 50 °C, and then digested in 1 ml of conc. HNO3 at 70 °C for 2 h. The digested samples were then diluted with 5 ml ultrapure deionized water for analyzing heavy metals (Federici et al. 2007).
3. The bioaccumulation factor (BAF), which is the ratio of the chemical concentration in an organism or biota to the concentration in water (Gobas and Morrison 2000), was calculated as follows:
  $$\textrm{Bioaccumulation}\ \textrm{factor}\ \left(\textrm{BAF}\right)=\textrm{concentration}\ \textrm{of}\ \textrm{the}\ \textrm{metal}\ \textrm{in}\ \textrm{snail}\ \textrm{tissues}\ \left(\textrm{mg}/\textrm{g}\ \textrm{dry}\ \textrm{weight}\right)/\textrm{concentration}\ \textrm{of}\ \textrm{the}\ \textrm{metal}\ \textrm{in}\ \textrm{water}\ \left(\textrm{mg}/\textrm{l}\right).$$

BAF < 1 indicates no contamination; 1 > BAF ≤ 10, the snail is tolerant; and BAF > 10, a hyperaccumulator (Ávila et al. 2017).

### Lab study

#### Snails

*Biomphalaria alexandrina* (Ehrenberg, 1831) snails (9–10 mm) were maintained in Medical Malacology Laboratory, Theodor Bilharz Research Institute (TBRI), Giza, Egypt. Snails were kept in plastic aquaria (16 × 23 × 9 cm) with dechlorinated aerated tap water (10 snails/l), pH: 7 ± 0.2, and temperature (25 ± 2 °C). Oven-dried lettuce leaves, blue-green algae (*Nostoc muscorum*), and TetraMin were provided to aquaria for feeding and 30 mg/l calcium carbonate (CaCO3) for snails’ shell and fecundity (Eveland and Haseeb 2011; Ibrahim and Bakry 2019).

#### Manganese (Mn) and exposure conditions (Mn)

A stock solution (1000 mg/l) of manganese (as MnCl_2_·4H_2_O, Fisher Scientific, Fair Lawn, NJ, USA) was made in distilled water.

### Bioassays

#### Miracidicidal and cercaricidal activities

Five milliliter of 87.5 mg/l of Mn solution was mixed with 5 ml of water containing about 100 freshly hatched miracidia or cercariae. As well as another 10 ml of dechlorinated tap water containing 100 freshly hatched miracidia or cercariae was kept as a control (Ibrahim et al. 2021). After intervals of 30, 50, 70, 90, 110, and 130 min, the alterations in the movement of miracidia and cercariae were observed under a dissecting microscope (Eissa et al. 2011).

#### Survival and infection rates

Laboratory juveniles *B. alexandrina* snails (4–7 mm) were exposed individually to 4–6 freshly hatched *S. mansoni* miracidia for 24 h and then transferred to separate aquaria. From day 21 post-exposure, each snail was tested weekly for the shedding of cercariae by exposing it to fluorescent light in 1 ml of water for 1 h at 25 °C (Hung et al. 2015).

#### Toxicity on snails

Snails were subjected to 87.5 mg/l of manganese solution for either 24 h or 48 h followed by 1 day of recovery. For each exposure time, 30 adult snails (9–12 mm in shell diameter) were used in three replicates, each of 10 snails/liter. The control group was also set up in triplicates using only distilled water (WHO 1965).

##### Hemolymph collection and light microscopy preparation

The hemolymph of the living snails from each group was collected according to Nduku and Harrison (1980). Total hemocyte count was done by a Bürker-Turk hemocytometer (Der Knaap et al. 1981). Blood smears were done as monolayers to show the different shapes of the hemocytes (Ibrahim et al. 2018). To measure the phagocytic index, hemocyte suspension of 100 μl collected from each specimen was smeared on glass slides and incubated with activated charcoal particles for 1 h at 37 °C in a humid chamber for cell adherence. The phagocytic index was calculated as per the following formula: Phagocytic index= Number of cells with phagocytized charcoal particle/ Total number of cells counted x 100. 

$$\textrm{Phagocytic}\ \textrm{index}=\frac{\textrm{Number}\ \textrm{of}\ \textrm{cells}\ \textrm{with}\ \textrm{phagocytized}\ \textrm{charcoal}\ \textrm{particle}}{\textrm{Total}\ \textrm{number}\ \textrm{of}\ \textrm{cells}\ \textrm{counted}}\times 100$$

Also, to measure the mortality index, cells were treated with 50 μl of 0.25% trypan blue dye solution for 5 min. Cells that have taken up the dye are dead. The percentage of blue-stained cells represents a mortality index (Guria 2018): Mortality index= Number of cells with blue stained cytoplasm/ Total number of cells counted x 100. 

$$\textrm{Mortality}\ \textrm{index}=\frac{\textrm{Number}\ \textrm{of}\ \textrm{cells}\ \textrm{with}\ \textrm{blue}-\textrm{stained}\ \textrm{cytoplasm}}{\textrm{Total}\ \textrm{number}\ \textrm{of}\ \textrm{cells}\ \textrm{counted}}\times 100$$

##### Tissue preparation

From each treated group and the control group, soft tissues of snails were withdrawn from the shell using forceps, weighed (1g tissue/10 ml phosphate buffer) and then homogenized by a glass Dounce homogenizer. The homogenates were centrifuged at 3000 rpm for 10 min, and the supernatants were stored at −80 °C until used.

##### Investigation of testosterone and estradiol hormones

Hormone concentrations (T and E) were assayed for all groups according to the manufacturer instructions of T EIA kit (Enzo Life Science, MI, USA, ADI-900-065) and E EIA kit (Cayman Chemical Company, MI, USA, item no. 582251).

##### Investigation of the antioxidant responses SOD, the oxidative stress marker (malondialdehyde (MDA)), and total antioxidant capacity

The supernatant of the soft tissue homogenate from each group was used. Biodiagnostic kits (Biodiagnostic Dokki, Giza, Egypt) were used for the determination of SOD (Damerval et al. 1986). Malondialdehyde (lipid peroxide) was done according to Ohkawa et al. (1979), and total antioxidant capacity was estimated by kit (Cat. No. TA 2513) (Koracevic et al. 2001).

##### Histological studies

Sections of the digestive and hermaphrodite glands of *B. alexandrina* snails of either control or exposed groups were done and stained with hematoxylin and eosin according to Romeis (1948).

#### Statistical analysis

Student’s *t*-test was used for comparing the means of experimental and control groups (Murray 1981). All biochemical measurements were presented as mean ± SD.

## Results

The present results showed that Mn heavy metal could accumulate in the tissue of *B. alexandrina*, *P. acuta*, and *H. duryi* snails. The bioaccumulation of Mn heavy metal in *H. duryi* tissue was higher than that in tissues of *P. acuta* and *B. alexandrina* snails (Table 1).

**Table 1:** Bioaccumulation of Mn heavy metal in *B. alexandrina* snails’ soft tissue and water samples collected from Abu Rawash, Giza

Snails	Conc in tissue (ppb)	Conc in water (ppb)	Bioaccumulation factor
*Biomphalaria alexandrina*	0.231	0.0875	2.64
*Physa acuta*	0.289	0.0875	3.302
*Helisoma duryi*	0.3114	0.0875	3.558

**Fig. 1:**
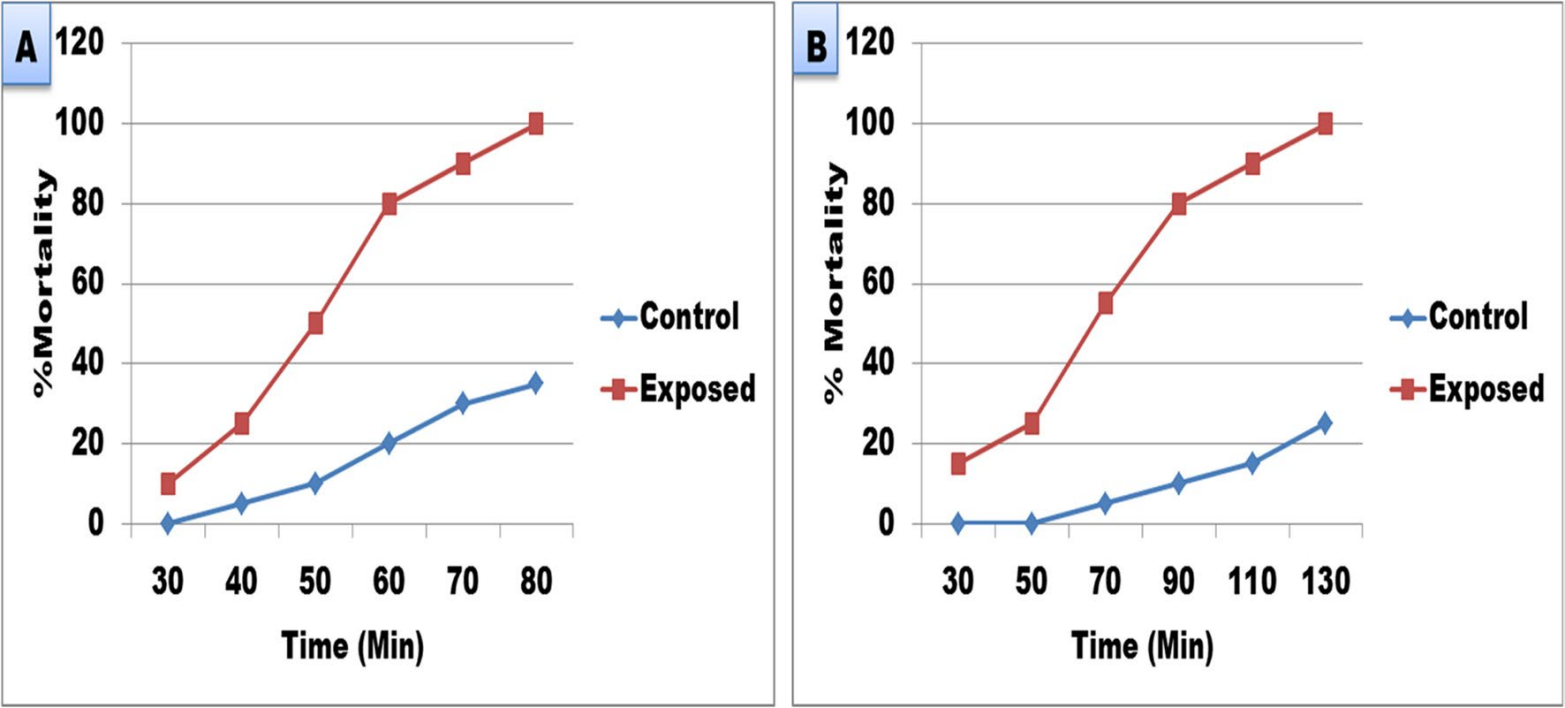
**A** Miracidicidal and **B** cercaricidal activities of Mn heavy metal (87.5mg/l)

The present results showed that Mn heavy metal (87.5 mg/l) has miracidial and cercaricidal activities, where after 80 min all miracidia had died compared to 35% deaths in the control group (Fig. 1). Also, all cercariae had died after 130 min of exposure compared to 25% of control group.

**Fig. 2:**
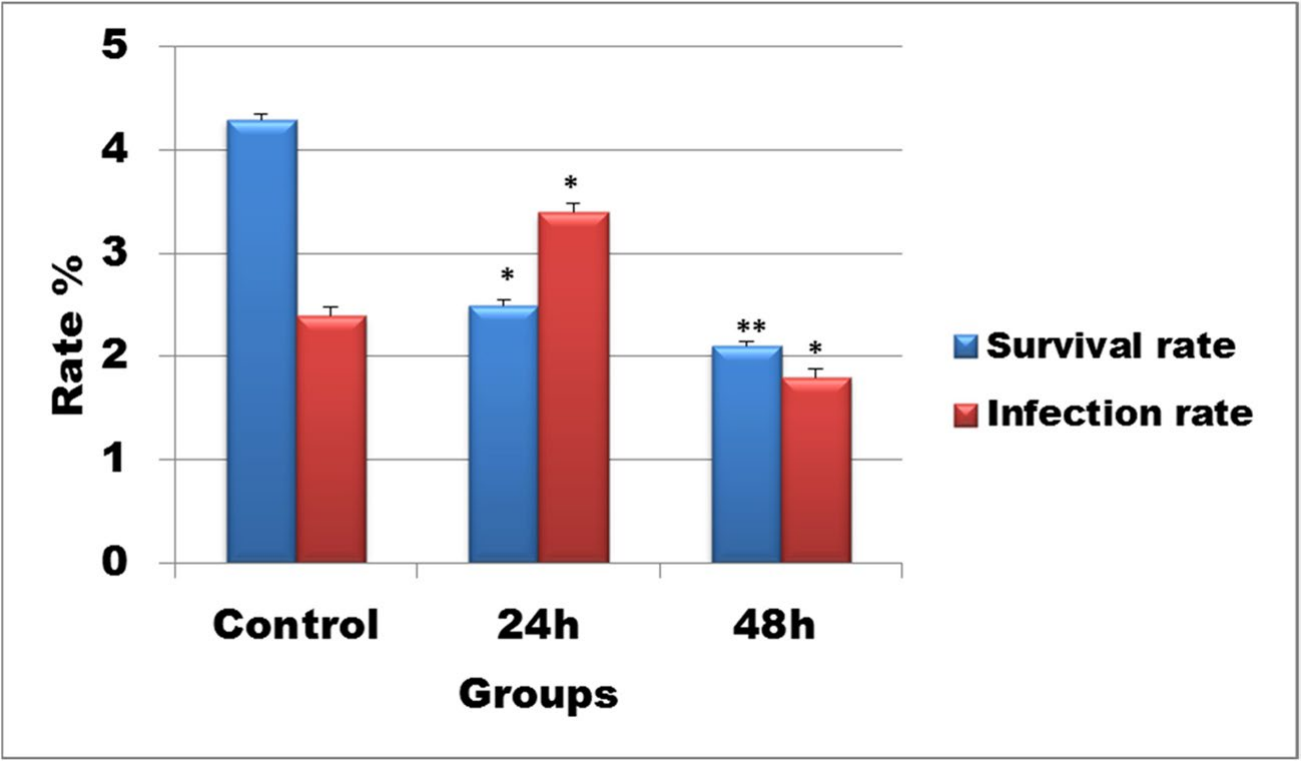
Histogram shows the effect of 87.5 mg/l Mn heavy metal exposure on survival and infection rates of *B. alexandrina* after 24 h or 48 h

The present results showed that after exposure of *B. alexandrina* snails to 87.5 mg/l Mn heavy metal for 24 h, the survival rate was significantly decreased, while the infection rate was increased. After exposure for 48 h, both survival and infection rates were significantly decreased (Fig. 2).

The present results showed that Mn heavy metal (87.5mg/l) significantly decreased the mean total number of the hemocytes (*p* ˂ 0.05) after exposure for 24 h or 48 h compared with *B. alexandrina* control group, while it significantly increased both the mean mortality index and the mean phagocytic index of the hemocytes of these snails (Table 2).

**Table 2:** Toxic effect of Mn heavy metal (87.5mg/l) on the total number of hemocytes/mm^3^, mean mortality, and mean phagocytic index of hemocytes of *B. alexandrina* snails

Groups	Hemocytes/mm^3^ ± S.D	% reduction	Phagocytic index	Mortality index
Control	648.33 ± 22.5		44.2	10
24 h	416.66 ± 28.86*	35.73	72.4	50
48 h	258.3 ± 38.18*	60.15	84.25	81.5

*Significant difference from control at *p* < 0.05

**Fig. 3:**
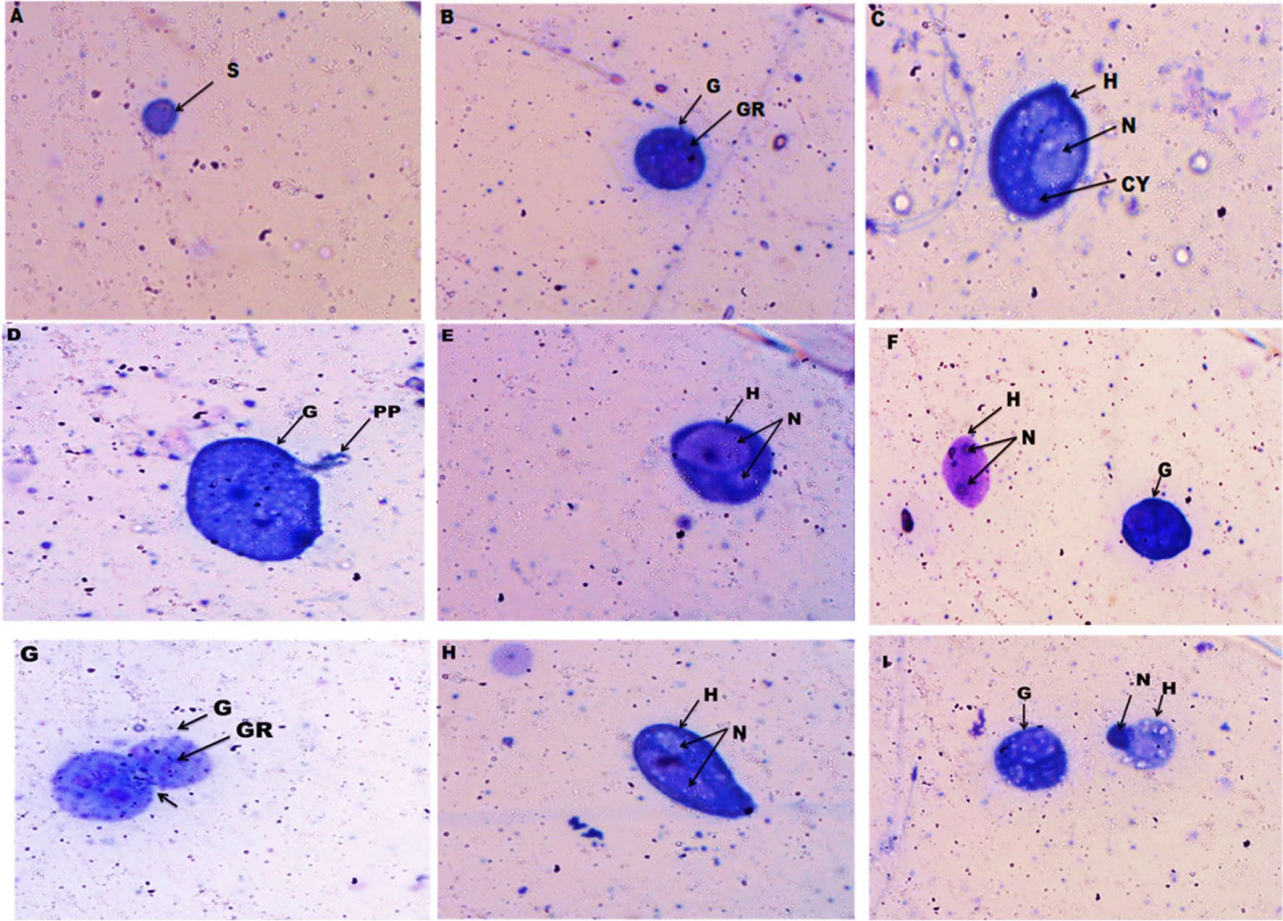
Photomicrographs show normal (control) hemocytes of adult *B. alexandrina* snails (**A**, **B**, and **C**) (×40). **D**, **E**, and **F**: After exposure to Mn for 24 h. **G**, **H**, and **I**: After exposure to Mn for 48 h. CY, cytoplasm; G, granulocyte; GR, granules; H, hyalinocyte; N, nucleus; PS, pseudopodia; S, round small. Arrow showed incomplete cell division

Examination of the hemocytes’ monolayers showed the presence of three shapes of *B. alexandrina* snails’ hemocytes in the normal control group: (a) small hemocytes, (b) granulocytes, and (c) hyalinocytes. After exposure to Mn heavy metal (87.5mg/l) for 24 h, the granulocytes had irregular cell membranes, forming pseudopodia and numerous granules. The nucleus of some hyalinocytes shrank and some had two nuclei divided in the same cell (Fig. 3D, E, F). After exposure for 48 h, some granulocytes had an irregular cell membrane with incomplete cell division, numerous granules, and forming pseudopodia. Hyalinocytes had a shrunk nucleus; others had two nuclei with irregular outer membranes (Fig. 3G, H, I).

The present results showed that both levels of testosterone (T) and estradiol (E) were significantly increased (*p* < 0.05) after exposure to Mn heavy metal (87.5 mg/l) for 24 h or 48 h compared with control group (Table 3).

**Table 3:** Effect of Mn heavy metal (87.5 mg/l) on T and E of *B. alexandrina* snails after 24 h and 48 h

Groups	Testosterone (nmol/L)	Estradiol (pg/ml)
Control	18.6 ± 0.2	96.8 ± 4.3
24	29.1 ± 0.1*	250 ± 4.2*
48	36.3 ± 0.6**	450 ± 5.4**

*Significant difference from control at *p* < 0.05

**Significant difference from control at *p* < 0.01

Exposure of *B. alexandrina* snails to Mn heavy metal (87.5 mg/l) to either 24 h or 48 h exhibited a significant increase (*p* < 0.05) in MDA and TAC contents compared with the control group. On the other hand, tissue SOD was significantly (*p* < 0.05) decreased after exposure in a time-dependent manner (Table 4).

**Table 4:** Effect of Mn heavy metal (87.5 mg/l) on MDA, SOD, and TAC of *B. alexandrina* snails

Parameters	MDA (nmol/g tissue)	SOD (U/g tissue)	TAC (mM/l)
Control	10.1 ± 0.12	9.13 ± 0.1	1.505 ± 0.4
24 h	12.12 ± 0.3*	5.2 ± 0.2*	1.88 ± 0.21
48 h	15.7 ± 0.13*	4.9 ± 0.32*	2.3 ± 0.13*

*significant difference from control at *p* < 0.05

**Fig. 4:**
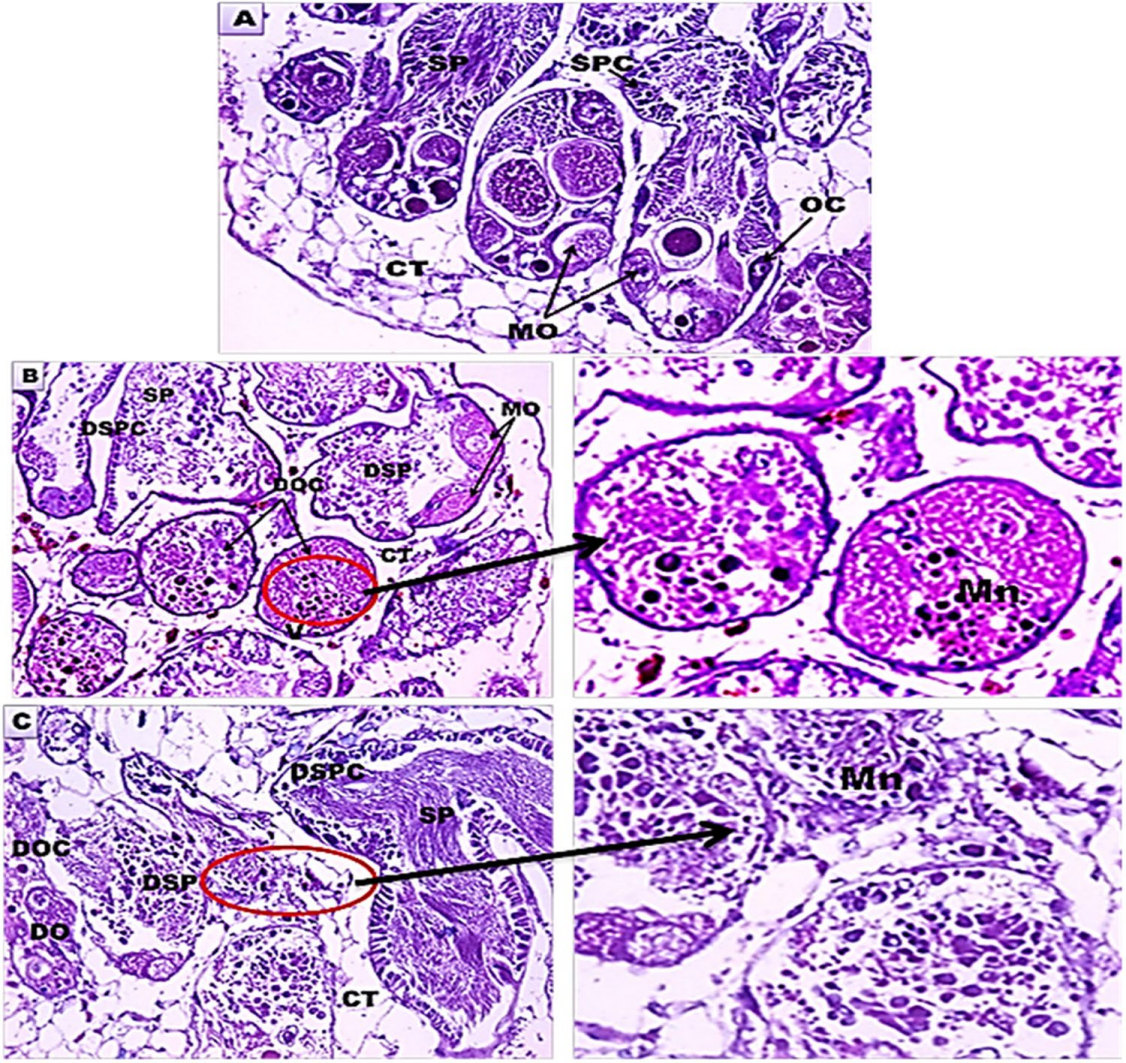
Light micrographs of *B. alexandrina* snails sections (H&E, ×40). **A** Control hermaphrodite gland. **B** Hermaphrodite gland after exposure to Mn heavy metal (87.5 mg/l) for 24 h. **C** Hermaphrodite gland after exposure to Mn heavy metal (87.5 mg/l) for 48 h. CT, connective tissue; DO, degenerated ova; DOC, degenerated oocytes; DSPC, degenerated spermatocytes; DSP, degenerated sperms; MO, mature ovum; OC, oocytes; SP, sperms; SPC, spermatocytes; V, vacuoles. The arrow pointed to Mn particles in the hermaphrodite acinus (×100).

The hermaphrodite gland of control *B. alexandrina* is composed of the male reproductive cells and the female oogenic cells. After exposure to Mn heavy metal (87.5 mg/l) for either 24 h or 48 h, there was a great damage in these gonadal cells including degeneration of sperms, some ova, oocytes, and spermatocytes. Also, the connective tissue between the acini was raptured, and the vacuoles were increased (Fig. 4A, B, and C).

**Fig. 5:**
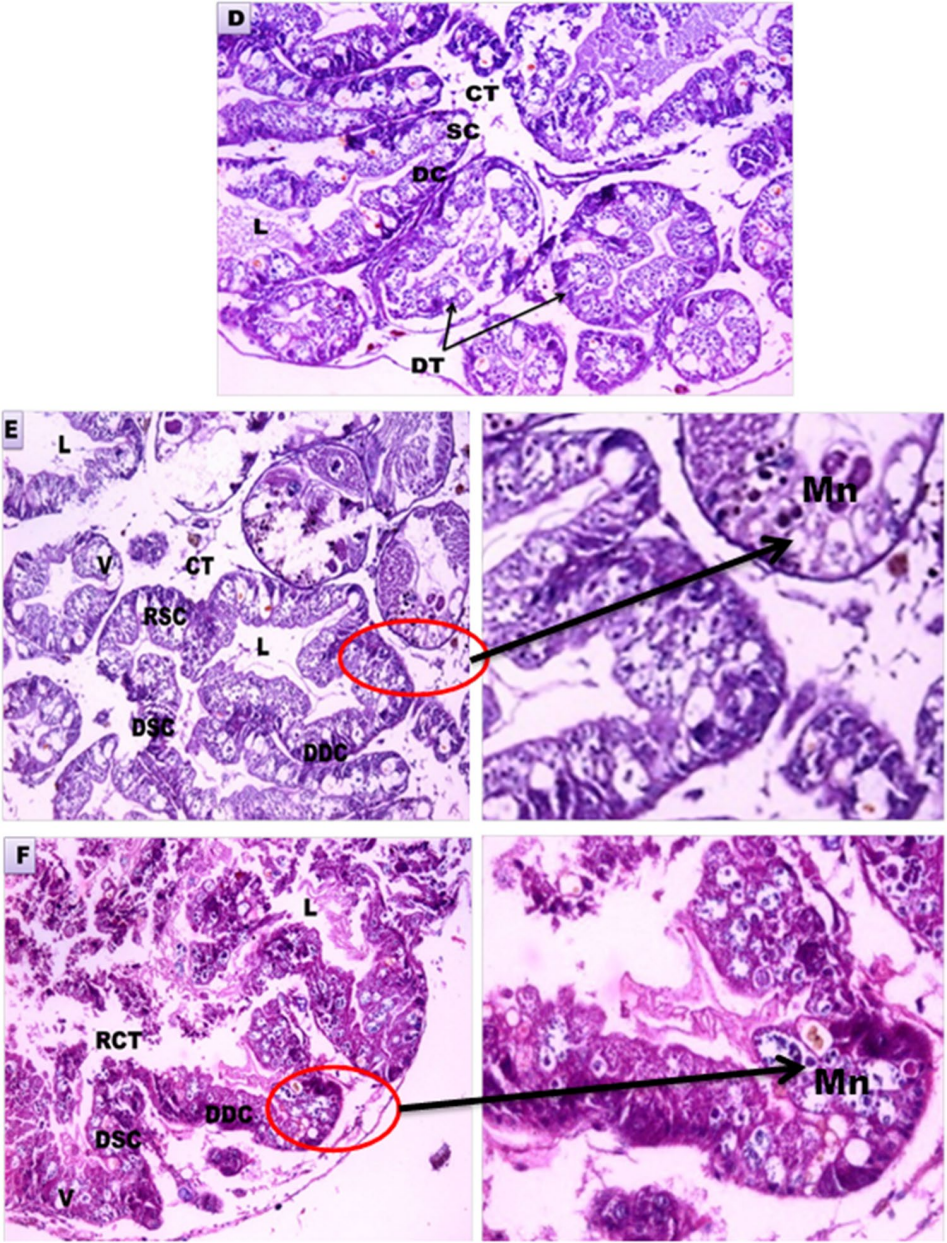
Light micrographs of *B. alexandrina* snails sections (H&E, ×40). **D** Control digestive gland. **E** Digestive gland after exposure to Mn heavy metal (87.5 mg/l) for 24 h. **F** Digestive gland after exposure to Mn heavy metal (87.5 mg/l) for 48 h. CT, connective tissue; V, vacuoles; DC, digestive cells; DDC, degenerated digestive cells; DSC, degenerated secretory cells; DT, digestive tubules; L, lumen; RCT, ruptured connective tissue; RSC, ruptured secretory cells; SC: secretory cells. The arrow pointed to Mn particles in the digestive tubule (×100)

The tubules of the digestive gland of normal *B. alexandrina* snails were lined by two types of cells, the digestive and secretory cells, and in between, there is the connective tissue. After exposure to Mn heavy metal (87.5 mg/l) for either 24 h or 48 h, there were great deleterious rupture and degeneration of the digestive cells and the secretory cells. Also, the vacuoles and lumen inside the tubules were increased, and the connective tissue was dissolved and ruptured (Fig. 5D, E, and F).

## Discussion

Heavy metal pollution has gained a great attention in research because of its dangerous consequences. The present results showed that the concentration of Mn heavy metal in water was 87.5 mg/l. This obtained concentration is higher than the allowable FAO limits for manganese metal (0.20 mg/l) (Simmons 2000). The present results showed that Mn heavy metal could accumulate in the tissue of *B. alexandrina*, *P. acuta*, and *H. duryi* snails. The bioaccumulation of Mn heavy metal in *H. duryi* tissue was higher than that in tissues of *P. acuta* and *B. alexandrina* snails. These results were in good accordance with Abdel Kader et al. (2016) who found that *P. acuta* and *H. duryi* were the highest snails that could accumulate heavy metals in their tissue and reasoned the high variation in BAF values depended on the type of the snail and the metal. The present results showed that BAF is higher than one in the three snails type and this indicated that these snails were tolerant (Liao et al. 2003; Ávila et al. 2017; Kachenga et al. 2020).

The present study elucidated the effect of 87.5 mg/l Mn exposure on different parameters of *B. alexandrina* snails. Results showed that this concentration of Mn (87.5 mg/l) has miracidial and cercaricidal activities, where after 80 min all miracidia had died and 130 min of exposure all cercariae had died. Cercariae and miracidia might be used as excellent indicator organisms for heavy metal pollution (Morley et al. 2003b). Cross et al. (2001) studied the effect of 4 mg/l manganese in artificial seawater on the cercariae of the marine trematode *Cryptocotyle lingua* and showed that this concentration induced reductions in cercarial quality which might lead to failure of transmission of this disease and concluded that the metal pollution could alter parasite populations and communities. Also, Morley et al. (2003a) found that the survival of *S. mansoni* exposed to Cd/Zn mixtures was significantly reduced at concentrations of 10 mg/l or higher. The authors reasoned this reduction in the effect of these metals on the enzymatic function of miracidia and leading to their death. These results could explain the decrease in the survival and infection rates of adult snails in the present study. Where, after exposure of snails to 87.5 mg/l Mn heavy metal for 24 h, the survival rate was significantly decreased, while the infection rate was increased. While, after exposure to 87.5 mg/l Mn heavy metal for 48 h, both survival and infection rates were significantly decreased. Similarly, Morley et al. (2003a) found that the survival rate of the infected *Lymnaea peregra* and *L. stagnalis* snails was severely reduced by exposure to cadmium metal.

Snails have an open circulatory system that contains the hemolymph, and it acted as the defense system against the foreign particles (Baroudi et al. 2020). The present results showed that Mn heavy metal (87.5 mg/l) significantly decreased the mean total number of the hemocytes after exposure for 24 h or 48 h compared with *B. alexandrina* control group. This decrease could be due to the sharing of these hemocytes in repairing the damages that occurred by Mn in both hermaphrodite and digestive glands (Esmaeil 2009; Ibrahim et al. 2018).

The main cellular component of the snails’ immune system is the hemocytes which are responsible for phagocytosis (Baroudi et al. 2020). The present investigation showed that Mn heavy metal (87.5 mg/l) significantly increased both the mean mortality index and the mean phagocytic index of the hemocytes of exposed snails. The increase in the mortality index is an indication of cell death or apoptosis (Guria 2018). This increase depends on the nature and the type of the stressors (Ibrahim and Sayed 2020).

Three types of hemocytes were found in smears of *B. alexandrina*. The more reactive cell was known as granulocytes, followed by small undifferentiated and then hyalinocytes that were found at the site of wounds (Ibrahim and Abdel-Tawab 2020). After exposure to Mn (87.5 mg/l) for 24 h, the granulocytes had irregular cell membranes, forming pseudopodia and numerous granules. The nucleus of some hyalinocytes shrank, and some have two nuclei divided in the same cell. After exposure for 48 h, some granulocytes had irregular cell membranes with incomplete cell division, numerous granules, and forming pseudopodia. Hyalinocytes had a shrunk nucleus; others had two nuclei with irregular outer membranes. Guria (2018) concluded that the alterations in the cytomorphology of hemocytes of *Lamellidens marginalis* (Bivalvia: Eulamelli branchiata) were due to the toxic effect of lead (Pb) heavy metal. The formation of pseudopodia of the granulocyte after exposure was a way to remove the foreign materials through the phagocytic process (Ibrahim et al. 2022).

Exposure to heavy metals causes deleterious health effects in the liver, kidney, brain, reproductive system, and other body systems. It impaired the hormonal regulation, steroidogenesis, and gametogenic process which is known as an endocrine disruptors (Verma et al. 2018). The present results showed that both levels of testosterone (T) and estradiol (E) were significantly increased after exposure to Mn heavy metal (87.5 mg/l) compared with the control group.

The present study showed that exposure of *B. alexandrina* snails to Mn heavy metal (87.5 mg/l) to either 24 h or 48 h exhibited a significant increase in MDA and TAC contents, while SOD was decreased compared with the control group. Khalil et al. (2017) conclude that the altered activities of SOD, CAT, GPx, GST and GR, and MDA levels could be useful biomarkers of water pollution with the heavy metals. These results in accordance with Siwela et al. (2010) who reported that the bioaccumulation of heavy metals in *Lymnaea natalensis* tissues increased malondialdehyde (MDA) levels, while decreased SOD and catalase (CAT) activities and reasoned these alterations due to the metal-induced oxidative stress. Also, Atailia et al. (2016) concluded that exposure of the terrestrial land snail *Helix aspersa* to metals under field and laboratory conditions caused changes in the non-enzymatic and enzymatic oxidative biomarkers, where it increased the activity of catalase and lipid peroxidation, while decreased GST activity and GSH level.

The present results showed that exposure of *B. alexandrina* snails to Mn heavy metal (87.5 mg/l) for either 24 h or 48 h caused great histopathological damages in both hermaphrodite and digestive glands. The damage in the gonadal cells included degeneration of sperms, some ova, oocytes, and spermatocytes with vacuoles and ruptured connective tissue between the acini. Also, there were great deleterious ruptures and degeneration of the digestive cells and the secretory cells. Also, the vacuoles and lumen inside the tubules were increased, and the connective tissue was degenerated.

These histopathological alterations might be related to the direct toxic effects of the heavy metals on the edible organs of target animals like fish (Elwasify et al. 2021; Morad et al. 2023), where the metal caused vacuolated hepatocytes, dilated central veins, compressed blood sinusoids, and congestion were seen in fish liver. Similarly, Abdel-Tawab et al. (2022) reported that cerium oxide nanoparticles synthesized with *Moringa oleifera* seeds at a concentration of 314.5 mg/l caused great damages in both hermaphrodite and digestive glands. From the previous result, it was noticed that the manganese heavy metal affects negatively many of the biological activities of *B. alexandrina* snail. So it is important to estimate the quantity of such metals in freshwater snails to determine snails’ ability to be bioindicators for these metals in the aquatic environment.

## Conclusion

The results demonstrated that manganese metal had a significant effect on *B. alexandrina* snail causing great damage and elevating the levels of testosterone and estradiol as well as other biological and physiological factors such as increasing the mean mortality and phagocytic index of the hemocytes. Additionally, it has an impact on *S. mansoni* larval stage activities that control disease transmission. As a result of their capacity to accumulate these toxins in their tissues, *B. alexandrina* snails could be employed as sensitive heavy metal pollution bioindicators.
